# The Latest Work of the British Red Cross Society

**Published:** 1912-06-22

**Authors:** 


### THE ROYAL ARMY MEDICAL CORPS SECTION.

### The Latest Work of the British Red Cross Society.

We have in previous issues of The Hospital made reference to the very creditable work done by the British Red Cross Society in the formation of Voluntary Aid Detachments for service with the Territorial Medical Service, and there are many indications that the movement still continues popular, for one of the recent returns shows that up to February of this year no less than 1,318 detachments had been registered at the War Office, representing a *personnel* of close on 40,000 women and men enrolled in the United Kingdom for service with the Territorial Medical Force in case of invasion.

Since then, no doubt, additional detachments have been raised, and the probability is that at the present time we have quite 50,000 women and men in our midst who are capable of rendering civilian aid to our sick and wounded Territorial soldiers should the occasion arise. This is a very satisfactory state of matters, and the country owes a debt of gratitude both to the members of the Voluntary Aid Detachments for coming forward as they have done, and to the British Red Cross Society for its public spirit in raising and organising them. Notwithstanding all this, we are inclined to suggest that the time has come when the whole question of this Voluntary Aid Detachment movement, as at present carried on, should be reconsidered in the interests of the British Red Cross Society.

### Voluntary Aid Detachments: A Criticism.

In the first place, the work is making in time of peace financial demands on the County Red Cross Committees to a considerable extent, and their funds are being heavily taxed to pay for work that should be paid for by the Territorial County Associations out of their Government grants, inasmuch as the Voluntary Aid Detachments fill a distinct gap in the Territorial Medical Service and are as necessary in the system of aid to the Territorial wounded as any of the field units. We feel satisfied that the Territorial Associations of the various counties would gladly give such monetary assistance if the consent of the War Office could be obtained, but so far this has been refused.

The truth is the initial error took place when the British Red Cross Society agreed to undertake the work of forming the detachments without first stipulating that the cost of organising them and of providing the necessary instructional equipment for them should be borne by the Government. It is now too late to remedy this mistake, but we think that a halt should be made in the formation of more detachments, or at least a plan devised whereby the detachments in a county should numerically bear a ratio to its annual Red Cross subscribers, each additional detachment above the ratio requiring the enrolment of sufficient new subscribers to represent an increase of £15 in the annual income. In this way the detachments would be kept within reasonable limits and there would be greater facilities for perfecting and improving those already in existence.

Necessary, however, as is the limitation of the detachments on financial grounds, we think that there is another and still more important reason for taking action in the matter, and it is this. The idea is becoming very prevalent that the British Red Cross Society has been established simply for the purpose of raising and training Voluntary Aid Detachments for the Territorial Medical Service, and that this constitutes Red Cross work, the result being that all the energy is being thrown into the detachments, while in other respects our counties are not being sufficiently organised to meet the demands that may be made upon them both in time of invasion and in the case of a foreign war. It would be very unfortunate for the future of the British Red Cross Society if such a view became universal, and we would urge on the British Red Cross Council the importance of taking the necessary steps to correct such an erroneous impression. This can best be done by no longer giving to the Voluntary Aid Detachments the prominence that has been so long bestowed upon them and by taking in hand the organisation of the counties for the real and varied duties embraced in the Red Cross work.

In our opinion what is really needed just now, in the interests of our Red Cross Society is to make it generally understood, and above all by the County Red Cross Committees, that this Voluntary Aid Detachment movement, although undertaken by the Society, scarcely falls within the scope of its duties, as it is really an integral part of the Territorial Force and should have been provided by the Government. In addition to this, decided steps should be taken to spread abroad a true knowledge and appreciation of the real work of our Red Cross Society.

### A Neglect of the County Committees.

The public should be instructed that this consists in giving *additional civilian aid* to our sick and wounded soldiers, both Regular and Territorial, in case of foreign wars and of invasion, and that this can only be efficiently and economically done by proper organisation in time of peace. A perusal of the Society's leaflet that describes the nature of the aid likely to be furnished by it in time of war either abroad, or at home in case of invasion, indicates very clearly the lines on which such organisation should proceed. It should take the form of making the County Red Cross Committees as comprehensive as possible and of forming from them sub-committees for dealing with the complete organisation of their individual counties in the matters of finance, of invalid transport, of additional *personnel* of doctors and nurses, of buildings suitable for hospitals, of convalescent homes, of clothing and hospital comforts, with arrangements for their proper packing and despatch, and of establishing small committees in every town and village in the county for dealing locally with these different points. It is only by such a sub-division of labour that method and system can be ensured, and that there can be prevented that overlapping and waste of energy that were so conspicuous in the civilian aid rendered during the South African War. The British Red Cross Society has been in existence now for some years, and it is because we feel that it has not developed on the practical and utilitarian lines that are necessary for its success in war that we venture to draw attention to the present position of matters. The County Red Cross Committees are not preparing for the real work of the Society, which is to be ready to serve as the channel through which the public can give that additional medical aid to our soldiers in time of war that it is always anxious to furnish, but they are devoting too much of their energies to one single movement and allowing it to overshadow everything else. We hope that our friendly criticism will not be lost sight of, and that active steps will be taken in the direction we have indicated.

